# Preliminary Air Quality and Microclimatic Conditions Study in the *Santuario della Beata Vergine dei Miracoli* in Saronno (VA)

**DOI:** 10.3390/molecules28041615

**Published:** 2023-02-07

**Authors:** Andrea Bergomi, Valeria Comite, Vittoria Guglielmi, Mattia Borelli, Chiara Andrea Lombardi, Roberto Bonomi, Concetta Pironti, Maria Ricciardi, Antonio Proto, Carlo Mariani, Paola Fermo

**Affiliations:** 1 Department of Chemistry, University of Milan, Via Golgi 19, 20133 Milano, Italy; 2 Scuola di Restauro ENAIP Botticino, Via Cosenz 54, 20158 Milano, Italy; 3 Department of Medicine, Surgery and Dentistry “Scuola Medica Salernitana”, University of Salerno, Via S. Allende, 84081 Baronissi, Italy; 4 Department of Chemistry and Biology, University of Salerno, Via Giovanni Paolo II 132, 84084 Fisciano, Italy; 5 Architect, Via Giusti 21/B, 20831 Seregno, Italy

**Keywords:** air quality, microclimate, cultural heritage, preventive conservation

## Abstract

In the present work, the microclimatic conditions (temperature (T), relative humidity (RH), and illuminance (I)), together with the air quality (both aerosol particulate matter (PM) and gaseous pollutants), were monitored to evaluate the environmental conditions inside the *Santuario della Beata Vergine dei Miracoli* in Saronno (VA), a masterpiece of the Italian Renaissance. For this purpose, dataloggers were used to carry out the T, RH, and I measurements, whereas an optical particle counter (OPC) was employed to perform the particle count and determine the concentration of the aerosol PM. Finally, diffusive passive samplers were used to determine the concentration of nitrogen dioxide (NO_2_) and BTEX (benzene, toluene, ethylbenzene, and xylenes). To identify possible spatial variations, the studies were conducted at different sites and different heights in the Sanctuary. Particular focus was given to the Easter week during which liturgical services attracting large numbers of people were carried out. Additionally, a comparison with the outdoor values was performed to highlight the accumulation phenomena and other variations in the concentrations of the species. Despite the indoor concentrations of pollutants and variations in the thermohygrometric parameters being generally lower compared to the outdoors (e.g., 5.2–15.0 µg m^−3^ versus 17.7–45.3 µg m^−3^ for NO_2_), the microclimatic conditions were often not in line with the Italian legislation and technical standards.

## 1. Introduction

In the last years, a topic of increasing concern among the scientific community regarding the conservation of cultural heritage is how to guarantee the optimal indoor conditions to safeguard a wide range of works of art stored in museum environments as well as historical archives [1,2,3,4]. 

The *Santuario della Beata Vergine dei Miracoli* was built between the 15th and 17th centuries following a miraculous event and is located in Saronno, a small town in the Lombardy region of Northern Italy. Once the architecture of the sanctuary was completed at the start of the 16th century, some of the most renowned and influential artists of the time were summoned to work on the interior decorations [5]. The most famous Lombard painter of that time, Bernardino Luini, decorated the apse and presbytery of the church with some masterpieces such as the *Marriage of the Virgin* while the dome was entirely frescoed by Gaudenzio Ferrari, another outstanding Lombard painter of the 16th century. In addition, two marvelous wooden sculptural groups, *Deposition* (1528–1529) and *Last Supper* (1531–1532), were carved by the sculptor Andrea da Corbetta and decorated and gilded by Alberto da Lodi [5]. 

Poor indoor air quality and microclimatic conditions are two factors that contribute significantly to the degradation of works of art such as the ones previously mentioned [4,6,7]. For this reason, museums have imposed concentration limits on the major air pollutants, along with temperature, relative humidity, and illuminance ranges that need to be respected [8,9]. However, the same regulations do not apply directly to sanctuaries and other indoor sites that attract large numbers of people, acting as vehicles for the penetration of pollutants from the outdoors [4,6]. Therefore, a proper and thorough air quality characterization is of the utmost importance for safeguarding works of art in such places.

Pollutants can directly damage artworks by causing yellowing or blackening phenomena, and due to their high reactivity they can accelerate degradation processes such as corrosion and oxidation [10]. It is important to consider that the damage caused by indoor air pollutants on museum objects is not always evident and obvious. Furthermore, pollutants can act in a synergic way with other factors (humidity, temperature, illuminance), and often the overall effect could be worse than that caused by any individual one [2]. 

While pollutants are emitted by both natural and anthropogenic sources in outdoor environments (e.g., in urban environments by fossil fuel combustion, biomass burning, industrial emissions, etc.) [11,12], in indoor environments, together with a contribution from the outdoors (due to air penetration), the works of art themselves can be responsible for pollutant emissions [13] (e.g., fossil specimens can release some toxic compounds used in conservation treatments) [14]. Often, the levels of internal air pollution, especially in urban environments, can easily reach the external pollution levels, particularly when appropriate air filtering systems are not used [15,16]. Furthermore, there is a wide range of pollutants that arise from specific indoor activities [17], building material emissions [14], or are due to the presence of visitors [3] who are responsible for particle transportation. In this regard, indoor–outdoor ratios are a useful tool for establishing the likely sources of air pollutants within buildings [18]. 

The most harmful gaseous pollutants to cultural heritage are NO_X_, SO_2_, O_3_, and volatile organic compounds (VOCs) [10,19]. These pollutants mainly originate from outdoor sources, even if some indoor sources are often present [7,12]. These species are responsible for numerous negative effects on the objects stored in museum environments including, but not limited to, chromatic alterations, superficial deposits, and erosion [4,10]. NO_X_ and SO_2_ are primary pollutants and originate mainly from traffic emissions and combustion processes [12]. In contrast, ozone (O_3_) is a secondary pollutant that is formed predominantly in polluted areas following the reaction between molecular oxygen (O_2_) and atomic oxygen (O), which in turn is generated by the photolysis of nitrogen dioxide [20]. Instead, volatile organic compounds represent an extremely diverse class of compounds, both of primary and secondary origin, with numerous outdoor and indoor sources [21]. Amongst the VOCs, BTEX (benzene, toluene, ethylbenzene, and xylenes) are the compounds that are usually found in greater concentrations, especially in highly polluted areas [22]. They typically share common sources, the most important being combustion processes and industrial emissions [23]. 

An additional risk factor for the objects preserved in museums is represented by aerosol PM [6]. Particles dispersed in the atmosphere can be of variable sizes in an interval that can range from a few nanometers to tens of microns [17]. The more common fractions that are normally measured outdoors are PM10 and PM2.5 (particles with an aerodynamic diameter of less than 10 and 2.5 microns, respectively). The ultrafine fraction, on the other hand, consists of particles with a diameter of less than 100 nm. The hazards linked to the particles are dependent not only on their concentration (expressed as µg m^−3^), but also on their chemical composition and their size [24,25]. Normally, air quality monitoring takes place outdoors (cities, background and rural sites, remote sites, etc.); nevertheless, more recently, it has become clear that pollutant monitoring should also be carried out in museum environments. Worrying sources of pollution can be present inside the museum and can be exacerbated by outdated air circulation systems, penetration, and accumulation from the outdoors [2,3].

Internationally, many museum institutions have established internal protocols that, although representing an important reference, are not necessarily accepted and implemented in all contexts. Following numerous studies on air pollution, the threshold limits or maximum exposure levels to harmful pollutants have been assigned for outdoor environments. Indeed, the pollutant concentration limits are regulated for ambient air because of the negative effects of air pollution on human health. The European Union has developed an extensive body of legislation that establishes the standards and objectives for several pollutants in the air. In particular, the EU’s air quality directives (2008/50/EC Directive on Ambient Air Quality and Cleaner Air for Europe and 2004/107/EC Directive on heavy metals and polycyclic aromatic hydrocarbons in ambient air) set the pollutant concentration thresholds that must not be exceeded in a given period of time. In contrast, there are no limits regarding indoor air quality that must not be exceeded, and a unique internationally-accepted protocol does not exist yet. In general, guidelines and recommendations establish the basic criteria, giving indications and suggestions on the levels for some of the main parameters (e.g., T, RH, I, gaseous pollutants, and particulate matter) [13,26], but none of these must be enforced by law.

To define a standard regarding the methods of analysis and the assessment of environmental conditions suitable for the preservation of artifacts in their specific environment, the Italian Ministry of Cultural Heritage (MIBAC) has developed the D.M. 10 May 2001 “Guidance document on technical-scientific criteria and museum functioning and development standards”. This document is based on several scientific studies carried out from the first half of the 1980s and illustrates the recommended levels of the main pollutants (NO_2_, SO_2_, PM10, O_3_) and thermohygrometric parameters (T, RH, I) for the safeguard of the artifacts (Table 1). These values vary depending on the type and origin of the collection; nevertheless, the guidance document recommends avoiding abrupt daily variations and cyclical day–night variations. 

Moreover, in 1999, the Italian National Institution for Standardization published a document as part of the UNI 10829 rule “Goods of historical and artistic interest. Environmental conservation conditions. Measurement and analysis”, which was aimed at the conservation of artworks located in buildings specifically designed for this purpose. Once again, this technical standard indicates the recommended ranges for the main microclimatic variables (T, RH), with a focus on the average values and temporal gradients (Table 2).

Both documents focus on the idea of preventive conservation as a way to minimize restoration work and preserve the integrity of the artifact. Along these lines, the main goal of this study was to carry out a preliminary evaluation of the potential degradation risks within the Sanctuary. This was achieved by monitoring the concentrations of the main air pollutants (NO_2_, BTEX, and PM) and environmental parameters (T, RH, I) using appropriate instrumentation. By performing an annual monitoring campaign, a complete picture of the Sanctuary’s microclimate was achieved, highlighting the possible risk factors for the works of art and the importance of carrying out similar studies in all indoor sites hosting important artifacts, and not only in museums. 

## 2. Results

### 2.1. Thermohygrometric Parameters

**Figure 1:**
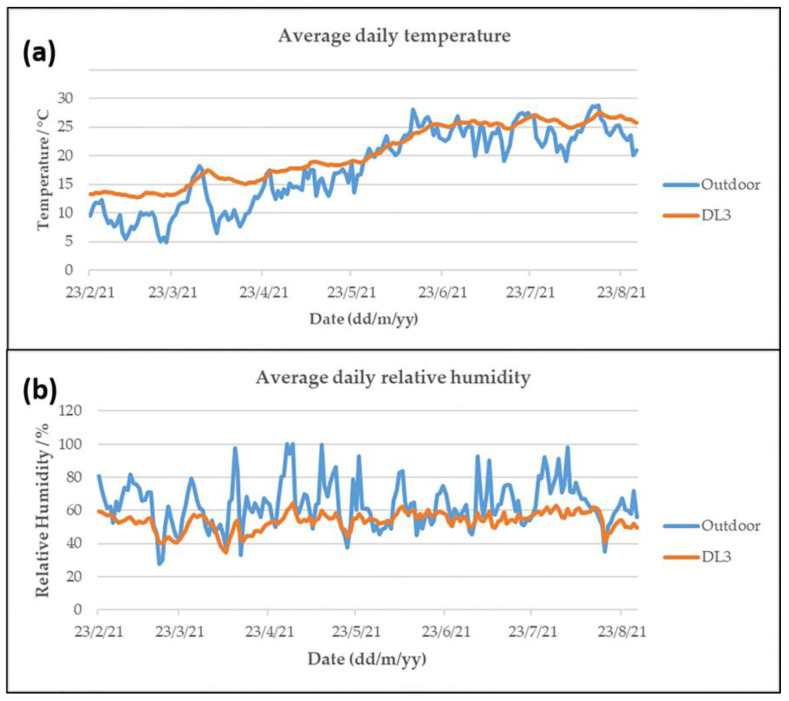
(**a**) The average daily temperature and (**b**) average daily relative humidity values reported for DL3 compared to the outdoor trends.

In Figure 1, the average daily temperature and relative humidity values are reported for DL3, along with a comparison with the outdoor values (ARPA Sensing Station, Saronno Santuario). Similar trends were observed for the other sampling sites and no significant differences in terms of the absolute values were found (Figures S1 and S2), indicating the presence of homogenous conditions within the Sanctuary. 

The trends observed in Figure 1 show a lower indoor temperature and relative humidity variability than outdoors. On the one hand, with a view to avoiding abrupt variations, the fact of not being significantly affected by external events is positive for the conservation of cultural heritage. However, compared to the recommended ranges and maximum values indicated in the UNI 10829:1999 technical standard, there were significant days in which these limits were overrun (Table 3).

These results highlight stable daily temperatures and greater daily relative humidity variations within the Sanctuary. Moreover, for both parameters, DL5 was associated with a greater number of days in which the respective limits were exceeded. This suggests an effect of the sampling height on the temperature and relative humidity variations, indicating more stable conditions on the ground and first floor of the church. On the one hand, considering that the limits apply to painted wood and not to wall painting, these conditions may represent only a partial problem for the church. On the other hand, the D.M. 10 May 2001 recommends avoiding abrupt variations of all thermohygrometric parameters, independently of the type of artifact under consideration, suggesting that these values may also represent an issue for the frescoes present in the Sanctuary.

Many overrun days were also observed for the absolute average temperature and relative humidity values. In these cases, the recommended ranges differed depending on the type of artifact under consideration. The temperature was highly dependent on the outdoor values (Figure 1), and therefore overruns were observed during the colder and the hotter months of the year. The window in which the temperatures complied with the values reported in the technical standard was very limited for painted wood and greater for wall paintings, as evidenced by the percentage days of overrun of more than 87% and less than 48%, respectively. Regarding relative humidity, once again, the ranges were different for the two types of artifacts considered, and in this case, a higher number of overrun days was observed for wall paintings as opposed to painted wood. Trends were not correlated with seasonality, as was the case for temperature, and overruns were observed randomly across all months of sampling. 

The D.M. 10 May 2001 suggests similar ranges for absolute temperature and wider ones for relative humidity compared to the UNI 10829:1999 technical standard (Table 1). With regards to temperature, the same percentage of overrun days would have been observed if the results were compared to the ranges of the Ministerial Decree. Instead, by making the same comparison, this percentage would have been lower for relative humidity. However, the average values observed in the monitored period also frequently fell outside the ministerial recommendations (Figure S2), confirming the fact that the thermohygrometric parameters are not controlled in the ideal way for the preservation of cultural heritage within the Sanctuary. 

Regarding illuminance, the Italian legislation places both wooden materials and frescoes under the same photosensitivity category (II, medium), and specifies a maximum illuminance of 150 lux. The results obtained for DL6 (*Last Supper)* and DL7 (*Deposition*) are displayed in Table 4. 

Both the maximum and average values did not exceed the indicated threshold and remained below 50 lux, which is the recommended limit for highly photosensitive materials such as silks and inks. Hence, the lighting levels within the Sanctuary are appropriate and do not represent a threat to the works of art. 

### 2.2. Particulate Matter

**Figure 2:**
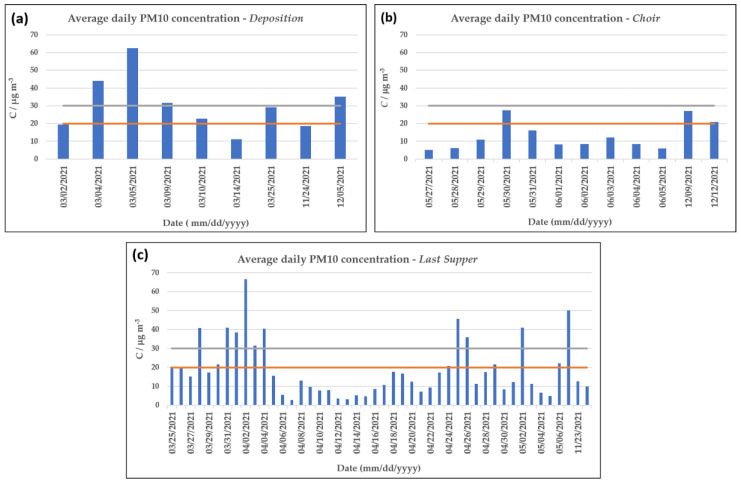
Average daily PM10 concentrations in the three sampling sites: (**a**) *Deposition*, (**b**) *Choir*, and (**c**) *Last Supper*. The orange and grey horizontal lines indicate the two maximum concentration limits indicated in the D.M. 10 May 2001.

Despite numerous sources stating that the fine fraction of PM is the most dangerous for the conservation of cultural heritage [25], the D.M. 10 May 2001 only states limits for the concentration of the coarser particles (PM10). Figure 2 shows the average daily concentration of PM10 detected at the three sampling sites compared to the limit (20–30 µg m^−3^) recommended by the ministerial decree.

For most of the monitored days, the PM10 concentration levels were below or within the specified range. However, occasional days of overrun were observed for the sampling sites in the two main later chapels, *Deposition* and *Last Supper*. Despite not performing the monitoring campaigns in parallel for the three sites, these preliminary results seem to suggest that particulate matter is mostly concentrated on the ground floor of the Sanctuary and is not transported quantitatively at greater heights. 

**Figure 3:**
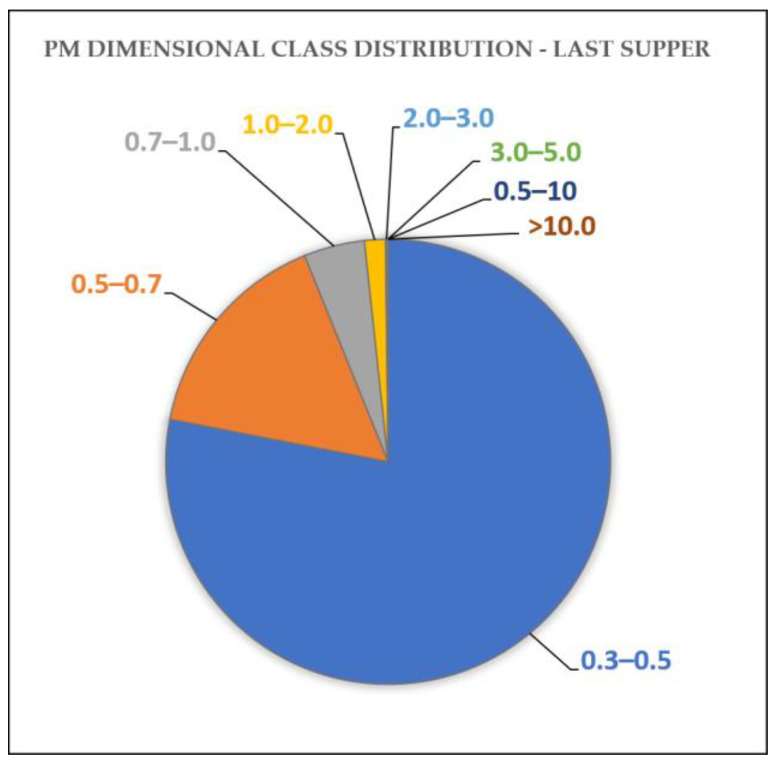
Dimensional class distribution of the particulate matter for the sampling site at the *Last Supper*. The ranges of the dimensional class are expressed in µm.

Thanks to the use of the optical particle counter, more detailed information regarding the dimensional speciation of the particles was obtained. As an example, the results relating to the *Last Supper* sampling site are reported in Figure 3, but similar values were also obtained for the other two sites (Figures S3 and S4). 

The results show the predominance of smaller particles (0.3–0.5 µm) and an overall decreasing contribution to the total number of particles with increasing size. This is also reflected in the mass concentration values since PM1 (particles with an aerodynamic diameter of less than 1 µm) almost always accounts for more than 50% of the mass of PM10 (Table 5).

Similar ratios between the concentrations of the three fractions were also observed at the other two sampling sites (Tables S1 and S2). Considering that, even if sporadic, daily average PM10 concentrations exceeding the 30 µg m^−3^ limit have been observed for both sites on the ground floor, the fact that the fine fraction accounts for most of these particles represents a potential threat to the works of art. 

**Figure 4:**
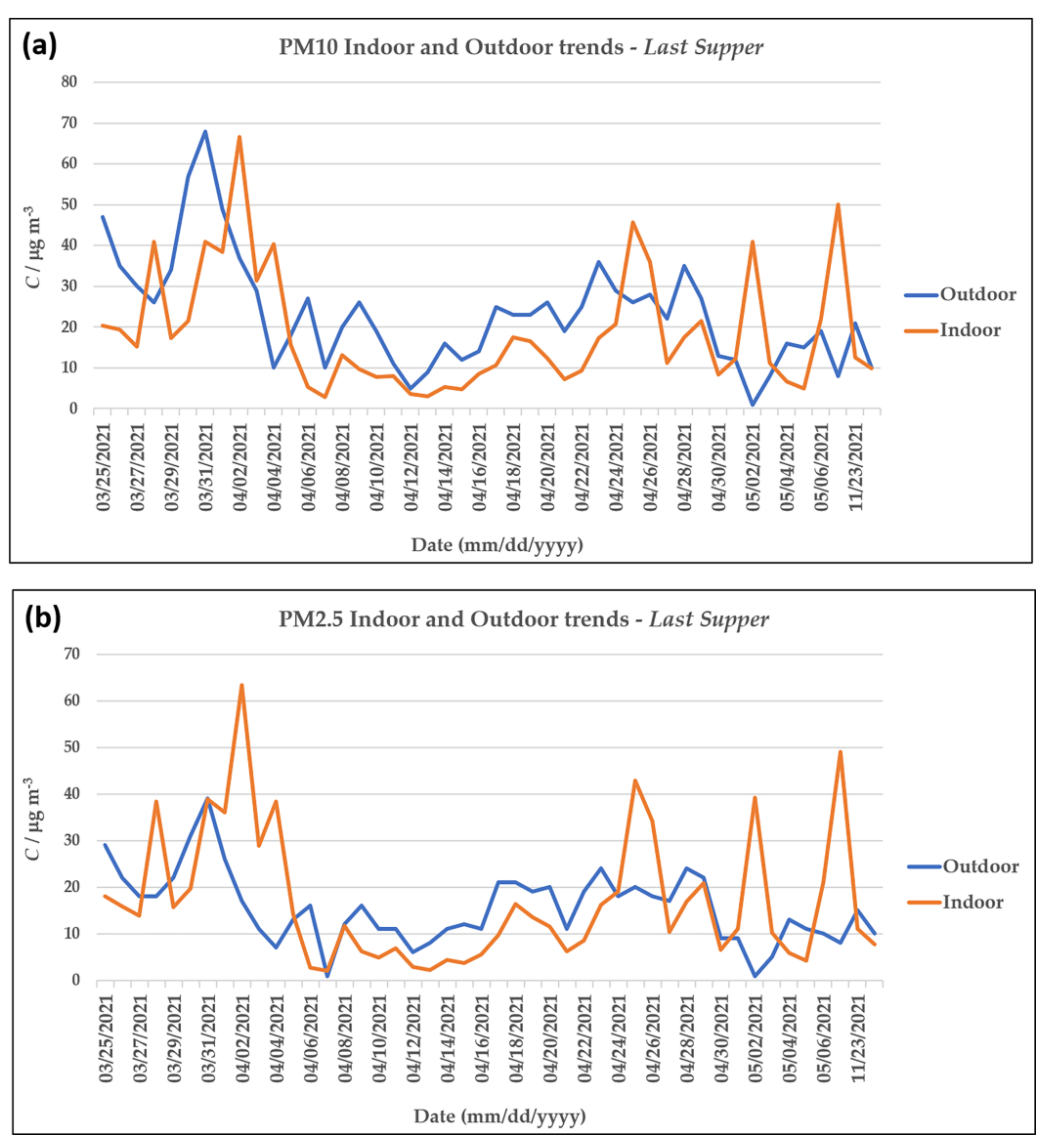
(**a**) The PM10 and (**b**) PM2.5 indoor and outdoor trends for the sampling site at the *Last Supper*.

In order to evaluate the origin and causes behind the presence of PM within the Sanctuary, the indoor concentrations were compared with the outdoor values (Figure 4). 

For most of the sampling periods, the indoor values followed the outdoor trends whilst remaining at lower concentrations, highlighting a shielding effect of the Sanctuary, which prevents the penetration of a fraction of the particles. However, occasional days in which the indoor values were higher than the outdoor ones were observed. Almost all these cases coincided with weekends or public holidays, which are known to attract a greater number of visitors and worshippers. Indeed, the sampling conducted during the Holy Week (28 March 2021–3 April 2021) highlighted numerous days in which the outdoor concentrations were overrun for both PM10 and PM2.5. 

**Figure 5:**
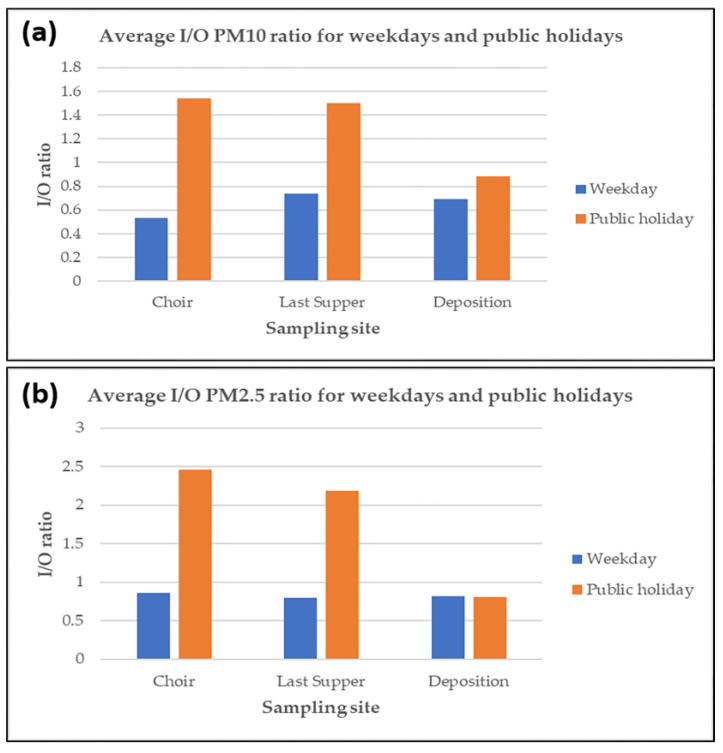
Average I/O ratio for (**a**) PM10 and (**b**) PM2.5 during the weekdays and public holidays for the three sampling sites.

Moreover, the indoor/outdoor (I/O) ratios were calculated for weekdays and public holidays (Figure 5). For the sampling sites at the *Last Supper* and *Choir*, a clear difference could be observed between the two different periods. The average I/O ratios were lower than 1 during weekdays, confirming a partial shielding effect of the Sanctuary, whereas they were higher than 1 during the public holidays, indicating the presence of specific sources to the days in question such as a higher influx of people, the use of candles, and incense burning. The effect was less pronounced for the sampling site at the *Deposition*; this was probably due to a minor impact of the sources on the days on which sampling was carried out for this site. Indeed, the monitoring campaigns were not carried out in parallel and the number of visitors and the use of candles and incense may have varied from day to day. 

**Figure 6:**
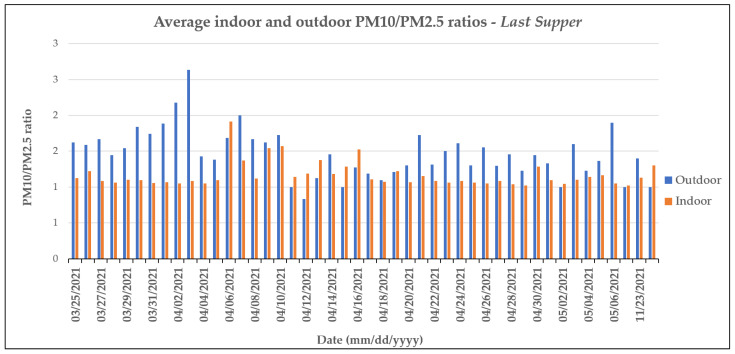
Average indoor and outdoor PM10/PM2.5 ratios for the sampling site at the *Last Supper*.

More in-depth analysis of the PM10 and PM2.5 values also enabled us to conclude that smaller particles are the ones that tend to accumulate indoors during public holidays and other festivities. Indeed, the PM10/PM2.5 ratios calculated for both the indoor and outdoor environments show that while these values were comparable during the weekdays, during the public holidays, the outdoor PM10/PM2.5 ratios were often higher than those indoors (Figure 6).

These differences were particularly evident at the *Last Supper* sampling site because the monitoring campaign was carried out partly during Holy Week, in which numerous festivities and religious ceremonies are concentrated. Indeed, the results showed that on the same days in which the indoor concentrations were higher than those outdoors, the difference between the PM10/PM2.5 ratio increased in favor of the outdoors. This suggests that the transport of larger particles from the outdoors to indoors is limited compared to the smaller ones, which tend to accumulate in closed spaces, leading to higher average indoor daily concentrations of particulate matter. 

### 2.3. Gaseous Pollutants (NO_2_ and BTEX)

The use of passive diffusive samplers allowed for the determination of the average NO_2_ and BTEX pollutant concentrations over the entire exposure period. Table 6 shows the results obtained for NO_2_ in the two studied time frames, the recommended values indicated in the D.M. 10 May 2001, and the average outdoor concentrations. 

As was the case for particulate matter, the NO_2_ concentrations were also lower indoors compared to the outdoors, once again highlighting a partial shielding effect of the Sanctuary. Despite this, the indoor concentrations registered were still higher than the limits of the Italian legislation, suggesting a problematic situation for the works of art. 

**Figure 7:**
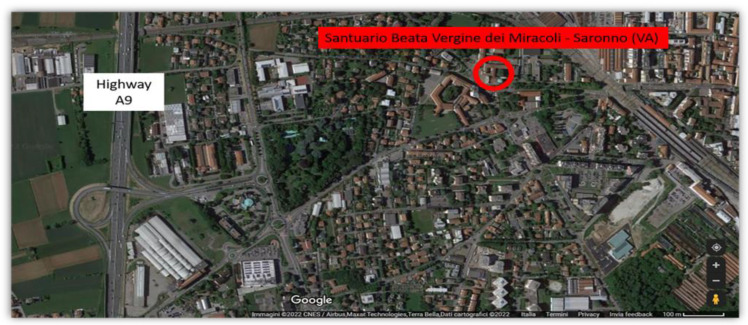
Location of the Sanctuary with respect to the A9 Highway.

Compared to nitrogen oxides, BTEX are a class of compounds that have not been extensively studied. The amount of data regarding their possible effects on cultural heritage, both in the literature and in legislative documents, is lacking. However, volatile organic compounds (VOCs) including BTEX are known to have multiple outdoor and indoor sources [22] and diagnostic ratios between the different species are useful to establish the most probable sources of pollution [21]. The preliminary results of this campaign show similar concentrations of benzene and toluene (1.6 and 1.7 µg m^−3^, respectively), while measurable amounts of ethylbenzene and xylenes were not observed. Similar concentrations of toluene and benzene are an indication of vehicular traffic as the main source of pollution [21]. This is not surprising considering the location of the Sanctuary, which is situated near the A9 Highway (Figure 7).

### 2.4. Preliminary Assessment of the State of Conservation of the Wooden Sculptures

**Figure 8:**
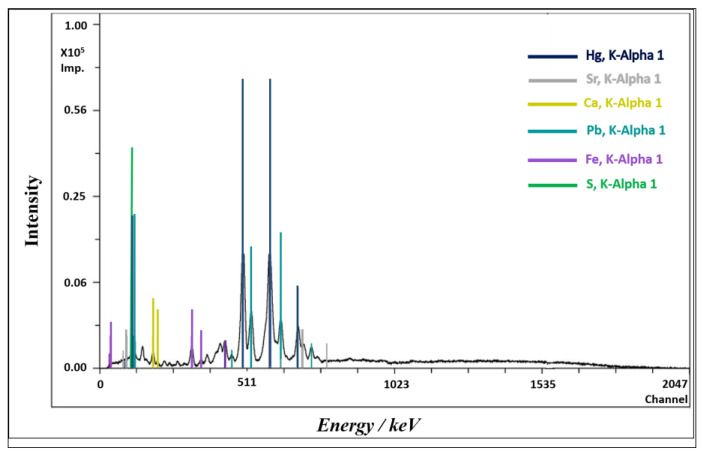
X-ray fluorescence spectrum of the wooden sculptural group.

**Figure 9:**
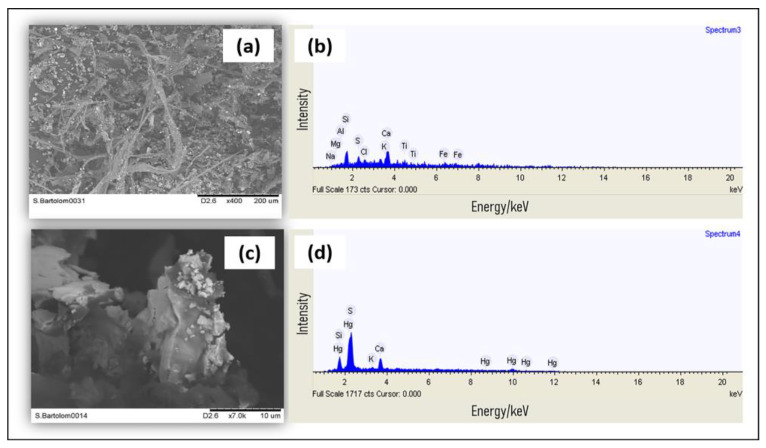
(**a**) SEM image of the dust deposited on the wooden sculptures (area = 0.80 mm × 0.60 mm, 400× magnification). (**b**) EDX spectrum of the image presented in (**a**). (**c**) Point image of the dust deposited on the wooden sculptures (Area = 0.04 mm × 0.03 mm, 7000× magnification). (**d**) EDX spectrum of the image presented in (**c**). Experimental parameters: accelerating voltage = 15 kV; working distance = 6500 µm; emission current = 65 mA; acquisition time = 150 s.

Preliminary analyses on the conditions of some of the wooden sculptures present in the two main chapels of the Sanctuary were carried out using X-ray fluorescence (XRF) directly on the works of art and scanning electron microscopy coupled with energy-dispersive X-ray spectroscopy (SEM-EDX) on the dust deposited on the sculptures. This enabled us to establish the presence of degradation phenomena originating from poor indoor air quality and microclimatic conditions. Indeed, the X-ray fluorescence spectra highlighted the presence of cinnabar (HgS) as the main pigment used to decorate the sculptures (Figure 8), and the same elements (Hg and S) were identified in the EDX spectra of the retrieved dust (Figure 9).

SEM analysis of the dust deposited on the wooden sculptures highlighted the presence of all the main constituents of atmospheric dust [27] including magnesium, sodium, calcium, chlorine, silicon, potassium, and iron. However, point analyses at greater magnifications enabled the detection of mercury, which is an element that is hardly ever found in concentrations above the instrumental SEM-EDX detection limits in atmospheric dust. The presence of this element is most certainly derived from the underlying substrate, which is represented by the wooden statue, highlighting the partial detachment of the pictorial film. 

The combined results of the two techniques indicate a poor state of conservation of the wooden sculptures. Considering that the powder was retrieved with the simple use of a brush, the fact that the same elements making up the substrate (identified thanks to the use of XRF) were also found in the deposited powder highlights the fragility of the artifact. The partial detachment of the pigment that was observed could be due to the chemical–physical interaction between the substrate and the deposited particulate matter, the degradation induced by the poor microclimatic conditions and air quality highlighted in the study, or a combination of both. 

## 3. Material and Methods

All of the sampling sites in which the campaign was conducted were chosen due to their proximity to the most important works of art of the Sanctuary. Special attention was given to the two main lateral chapels hosting the wooden sculptural groups of the *Deposition* and *Last Supper*, since these locations are potentially the most affected by different sources of pollutants. First, they are adjacent to the main altar where, during religious ceremonies, candles are lit and incense is burnt. Second, they are often the main attraction of weekly guided tours with numerous visitors and worshippers. Moreover, sampling at different heights was performed to evaluate the homogeneity of the conditions within the church. The specific monitoring periods for all the different parameters were determined in accordance with the Sanctuary officials and the availability of the desired sites. 

### 3.1. Thermohygrometric Parameters

Dataloggers were employed to monitor the temperature, relative humidity, and illuminance during the following period: 23 February 2021–28 August 2021. Specifically, USB Mini TH dataloggers (XS Instruments, Carpi, Italy) were used to measure the temperature and relative humidity. The measurement ranges were: −40/+80 °C for temperature (±0.5 °C (−40/−10) °C; ±0.3 °C (−10/+80) °C) and 0/100% for relative humidity (±3%). The resolution was 0.01 °C for temperature and 0.01% for relative humidity. HOBO U12-012 dataloggers (Onset Computer Corporation, Bourne, MA, USA) were used to measure the illuminance. The measurement range was 0–32,300 lumens m^−2^ (±2.5%) with a resolution of the external input channels of 0.6 mV. 

**Figure 10:**
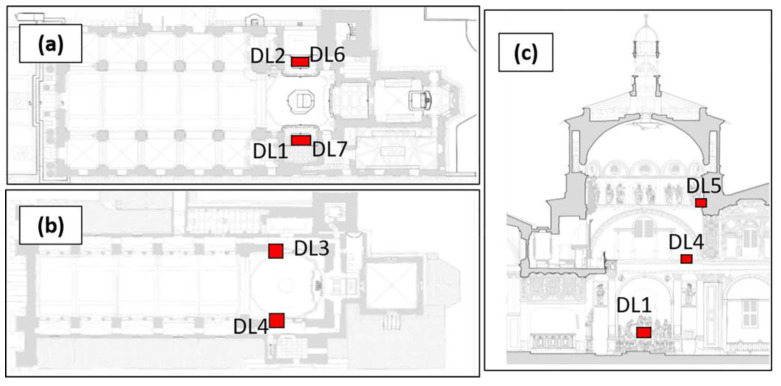
The floor plans and sections of the sanctuary showing the placement of the dataloggers: (**a**) Ground floor plan, (**b**) first floor plan, (**c**) right-side section (*Deposition*).

A total of seven dataloggers were used in this study (DL1-7), five measuring temperature and relative humidity (DL1-5), and two measuring illuminance (DL6-7). The instruments were placed in five different sampling sites and at three different heights in the Sanctuary (Figure 10). On the ground floor, dataloggers were placed close to the two main lateral chapels hosting the wooden sculptural groups *Last Supper* and *Deposition*. On the first floor, the instruments were positioned on the two ledges directly above the chapels, and on the second floor, one datalogger was placed on the side of the dome. Table 7 summarizes the locations and parameters monitored by each of the dataloggers.

The choice of the parameters in relation to the sampling site was based on the specific conservation issues of each location. Temperature and relative humidity are parameters that can vary with height, and therefore these parameters were monitored on three different floors of the Sanctuary. The presence of an LED lighting system at the two main lateral chapels (*Deposition* and *Last Supper*) required the monitoring of illuminance specifically at these sites. 

### 3.2. Particulate Matter

**Figure 11:**
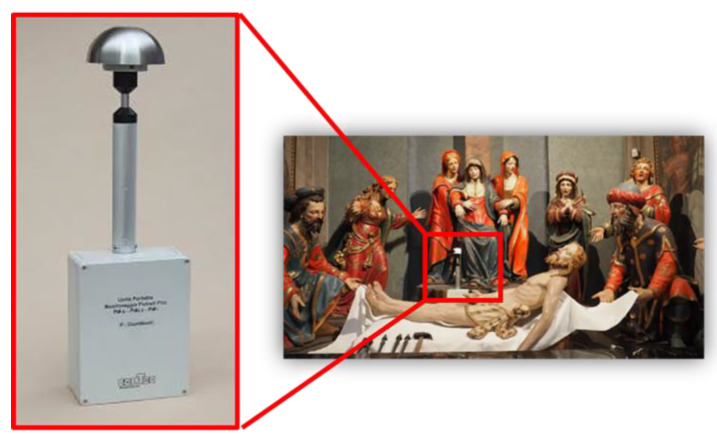
P-Dust Monit positioned in one of the main chapels close to the sculptural group of the *Deposition*.

An optical particle counter (P-Dust Monit, conTec Engineering Srl, Milano, Italy) was employed to monitor the particulate matter concentrations (Figure 11). 

The aerosol particles were aspirated with a constant-flow pump, which sucks in air through a radially symmetrical probe and conveys it into a chamber where they are individually hit by a laser light beam. The energy reflected by each particle, which is proportional to its size, is measured by a high-speed photodiode that outputs both the counting and dimensional characterization signals. The measurement sampling range was between 0 and 1000 µg m^−3^ with a sensitivity of 0.1 µg m^−3^. Measurements were performed in real-time with a detection every 60 s. 

**Figure 12:**
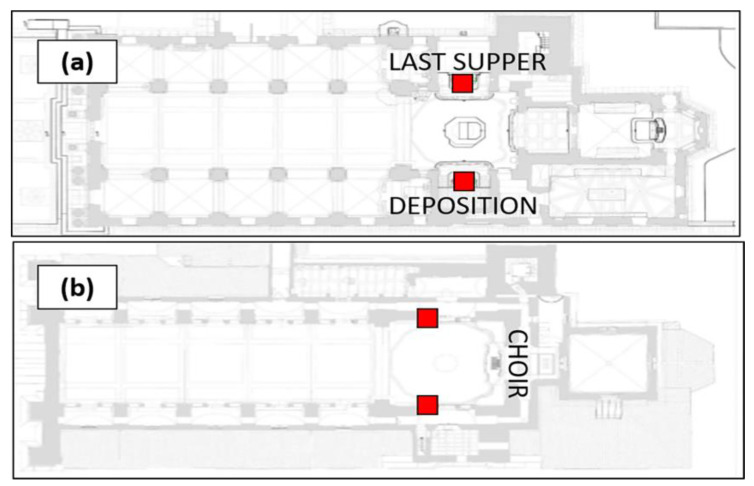
Planimetry of (**a**) the ground floor and (**b**) the first floor showing the three sampling sites.

The particles were classified into eight different dimensional classes (0.3–0.5 µm; 0.5–0.7 µm; 0.7–1.0 µm; 1.0–2.0 µm; 2.0–3.0 µm; 3.0–5.0 µm; 5.0–10 µm; >10 µm) and PM concentrations were expressed as PM10, PM2.5, and PM1. The campaign was carried out between 2 March 2021 and 12 December 2021, in which the P-Dust Monit was placed alternatively at three different sampling sites: the two main lateral chapels (*Deposition* and *Last Supper*) and the *Choir* on the first floor (Figure 12). 

For all the sites, monitoring was conducted during the weekdays, weekends, and public holidays. A longer period was monitored for the *Last Supper* site in order to evaluate the impact of the Holy Week (28 March 2021–3 April 2021) on the pollutant concentrations. One of the two main lateral chapels was chosen to carry out sampling during these festivities for the same reasons outlined in the opening paragraph of this section. 

### 3.3. Gaseous Pollutants (NO_2_ and BTEX)

**Figure 13:**
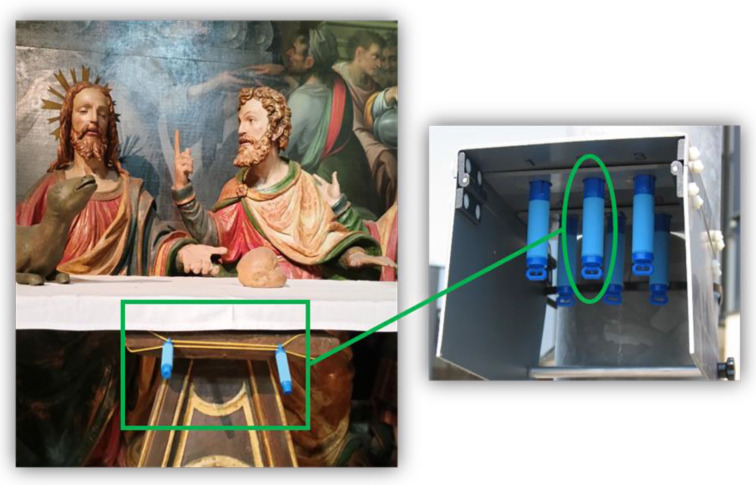
Diffusive passive samplers positioned in one of the main chapels close to the sculptural group of the *Last Supper*.

Passive samplers, RING^®^ radial diffusive devices purchased from Aquaria (Aquaria Srl, Milan, Italy), were used for pollutant sampling (Figure 13) according to NIOSH methodologies no. 1500 for BTEX and no. 6014 for NO_2_. The devices were positioned at the same sampling sites chosen for the monitoring of PM (Figure 3). Nitrogen dioxide was sampled from 23 March 2021 to 2 April 2021 (*Deposition* and *Last Supper*) and from 14 December 2021 to 28 December 2021 (*Deposition*, *Last Supper,* and *Choir*). Instead, BTEX were sampled from 23 March 2021 to 2 April 2021 (*Deposition*). 

### 3.4. Preliminary Assessment of the State of Conservation of the Wooden Sculptures

In order to further evaluate the microclimatic conditions within the Sanctuary, a preliminary assessment of the state of conservation of the wooden sculptures was performed through a series of non-invasive analyses. X-ray fluorescence (XRF) was performed directly on the artifacts with the aim to identify the constituent materials of the sculptures. Scanning electron microscopy coupled to energy-dispersive X-ray spectroscopy (SEM-EDX) was used to perform the morphological investigations and determine the elemental composition of the powder deposited on the works of art. The combined use of these techniques was employed to understand the possible interaction between the materials and the particulate deposit. Indeed, the evaluation of the chemical-physical interactions can reveal important information regarding the conservation status of the wooden sculptures.

XRF analysis was carried out using a Spectro xSORT portable XRF spectrometer (Spectro Analytical Instruments, Kleve, Germany) with the acquisition parameters as follows: current intensity: 50 µA; voltage: 40 kV; acquisition time: 60 s; spot diameter: 9 mm. Measurements were carried out by referring to the UNINormal 10705 “X-ray fluorescence analysis with portable instrumentation” and 10945 “Cultural heritage: characterization of pictorial layers. Generalities on analytical techniques used” technical standards.

The particulate material deposited on the sculptures was retrieved with the use of a brush. SEM-EDX analysis was performed with a TM4000PlusII Scanning Electron Microscope (Hitachi, Tokyo, Japan) coupled to an EDX microprobe. The images were obtained using back-scattered electron (BSE) mode in low vacuum conditions, and analyses of the selected point locations were also performed under the same conditions. 

## 4. Discussion

Museum objects should last for centuries or even millennia. Granted that degradation is an inevitable natural and progressive process, it can be accelerated by poor microclimatic conditions. Indeed, exposure to harmful pollutants and non-ideal thermohygrometric parameters, even if only slightly outside the recommended values, may cause substantial deterioration effects in the long run. Therefore, being able to conduct monitoring campaigns such as the one in this study is crucial in order to understand the conditions to which such works of art are exposed, evaluate the possible risks, and eventually act accordingly to prevent possible damage. This is often a challenging task considering the complexity and diversity of the artifacts that can be found on the same site, which renders the definition of the absolute optimal ranges and/or critical values for the proper conservation of cultural heritage particularly difficult. 

Indeed, in this study, it was not uncommon to observe days of sampling in which the microclimatic conditions in the Sanctuary were within the recommended values for painted wood but not for wall paintings, and vice versa. This was true for the temperature and relative humidity values, highlighting the difficulty in finding a balance between the proper conditions for one type of artifact and the other. However, the number of overrun days was above 29% for both parameters in terms of the average daily values, reaching values up to 97% (DL2, average daily temperature, painted wood). This suggests the presence of non-ideal microclimatic conditions inside the Sanctuary, regardless of the type of artifact under consideration. 

Regarding the particulate matter, the overall conditions in the church were less concerning, at least in terms of the number of days in which the limits were overrun. However, the indoor PM concentration values increased significantly during weekends and public holidays. One of the reasons behind this increase may be related to a larger influx of people, which is often associated with these festivities. In fact, several other studies have highlighted the role of visitors as vehicles for the transport of particles from the outdoors [4,6,7]. However, the same studies have indicated that visitors tend to favor the transport of larger particles (>1 µm) [6], whereas the results of this study seem to indicate the opposite. Other possible sources of particulate matter include the burning of candles and incense, which are regularly practiced during religious ceremonies. Indeed, other studies have shown that the concentrations inside churches can reach up to ten times the outdoor concentration values, which is particularly true for the finer fractions [28]. The indoor–outdoor differences observed in our study were less pronounced, probably due to the less extensive use of candles and incense; however, the impact on the overall indoor concentrations was still appreciable. 

Museums have already started to act on the issue of visitors acting as vehicles for the penetration of pollutants by putting safety measures in place such as restricted entries and ionization chambers [29]. These measures would certainly be more difficult to implement in the Sanctuary. As far as organized tours and visits are concerned, the possibility of limiting access and separating people into smaller groups could still be a viable option. However, the same cannot be applied to religious ceremonies such as the typical Sunday Mass, and alternatives for protecting these works of art must be found. 

The direct impact of visitors on the concentration of gaseous pollutants could not be observed in this study given the type of sampling system employed; however, an overview of the concentration of gaseous pollutants (NO_2_ and BTEX) was achieved. The average levels of the nitrogen oxides fell within the range of values observed in the literature (3–28.5 µg m^−3^) [7,30], but were always higher than the recommended values of the Italian legislation. This is certainly a potential risk for works of art since nitrogen oxides are known precursors of aggressive species such as nitric and nitrous acids [7]. A partial shielding effect of the Sanctuary was also observed for nitrogen oxides since the outdoor concentrations were always higher than the indoor ones. Despite this, a clear dependence on the outdoor pollutant levels was observed, since the indoor NO_2_ concentrations were higher during the winter campaign compared to the one carried out in spring. Moreover, no significant differences in terms of the ability to penetrate from the outdoors was observed for the different seasons. This may be because, unlike what occurs in museums, which tend to have greater pollutant penetration during the summer [6], the air exchange rate in churches does not vary significantly between the different seasons. 

With regard to BTEX, the results of this study confirm the limited penetration of pollutants from the outdoors, since the concentrations observed within the Sanctuary were lower than the typical outdoor values of similarly polluted areas [31]. On the one hand, the concentrations of benzene (1.6 µg m^−3^) and toluene (1.7 µg m^−3^) were lower than those found in some museum areas in Florence (1.4–2.8 µg m^−3^ for benzene and 13–35 µg m^−3^ for toluene) [19] and Naples (4.3–6.8 µg m^−3^ for benzene and 7–19 µg m^−3^ for toluene) [7]. On the other hand, these values were close to those observed in a small museum of Salerno (0.8–3.2 µg m^−3^ for benzene and 0.7–3.2 µg m^−3^ for toluene) [30]. Moreover, diagnostic ratios (toluene/benzene ratios) point to vehicular traffic as being one of the main sources of air pollution inside the Sanctuary. Therefore, despite previous results highlighting a limited penetration of pollutants, there is still a noticeable impact of outdoor sources on the air quality within the Sanctuary. 

Taking into consideration the results of the entire campaign, it is possible to conclude that the overall microclimatic conditions inside the Sanctuary represent a potential threat to the works of art. The use of appropriate sampling techniques and diagnostic methodologies was crucial in formulating this assessment. The use of dataloggers enabled the continuous monitoring of the thermohygrometric parameters, which was essential in order to establish the daily variations that were then compared to the normative references. Indeed, except for illuminance, all the monitored parameters were outside the specified ranges for the proper conservation of cultural heritage. The use of an optical particle counter also allowed for the continuous monitoring of particulate matter, which enabled the determination of concentration peaks that were then related to specific events occurring within the Sanctuary, and therefore the identification of indoor sources of pollution was possible. Moreover, the use of diffusive passive samplers enabled us to complete the evaluation of air quality by sampling NO_2_, which is one of the most aggressive and dangerous species for cultural heritage, and BTEX, which in turn enabled the identification of the main outdoor sources of pollution that also impacted the air quality within the church. Finally, the combined used of XRF and SEM-EDX was crucial in order to identify the degradation phenomena of the wooden sculptures such as the partial detachment of the pictorial film. 

Moving forward, the issue will be to find a way to control these parameters in an environment such as a Sanctuary. In recent years, museums have equipped themselves with HVAC (heating, ventilation, and air conditioning) systems in order to control the thermohygrometric parameters within the desired ranges to ensure the optimal microclimatic conditions for the works of art [32]. However, several limitations to these systems have been highlighted [33], and alternatives are currently being studied [34]. Their application to a place such as this Sanctuary, considering the dimensions of the building, would be a very difficult task, even without considering the cost of setting up these systems. Careful considerations will have to made in accordance with the local authorities in order to find the optimal solution for the protection of the works of art. The next stages of the work will include a second, more extensive, monitoring campaign. One of the future perspectives will entail the development and testing of new temperature, relative humidity, and illuminance sensors enabling the remote and real-time visualization of these parameters. This will allow for time-consuming operations such as the download and subsequent elaboration of data to be avoided and the immediate detection of values outside the recommended ranges. This could enable a quicker and more targeted identification of the events responsible for any overrun. Tests will also be conducted on new optical particle counters designed specifically for applications in the cultural heritage field. These devices monitor the same parameters, but are silent and smaller in size, and therefore of low visual impact. These characteristics make them easily adaptable in numerous settings without having to conceal parts of the work of art or disturb the visitors in any way. Moreover, continuous monitoring of the gaseous pollutants by employing advanced monitoring stations will be performed in order to evaluate the temporal concentration differences, which was not possible with the passive samplers employed in this study. Hopefully, once validated, all these systems will enable a complete spatial coverage of the Sanctuary, aiding in the enactment of targeted measures aimed at the conservation of cultural heritage.

## 5. Conclusions

Numerous studies during the last thirty years have highlighted the relationship between poor microclimatic conditions and the deterioration of works of art. Consequently, extensive monitoring campaigns have been conducted in environments hosting important artifacts, especially museums, and mitigations strategies are slowly being implemented. However, the research regarding alternative sites such as churches and sanctuaries, which in many cases contain works of art of historic and artistic interest, is lacking. 

With the aim to start filling this void, the current study focused on the determination of the microclimatic conditions and air quality within the *Santuario della Beata Vergine dei Miracoli*. An annual monitoring campaign was carried out measuring the temperature, relative humidity, and illuminance values, along with the particulate matter and gaseous pollutant concentrations. The results of this study highlight the poor microclimatic conditions within the Sanctuary, representing a potential threat for the conservation of the works of art located inside. Aside from the specific implications for the studied site, hopefully, this work will represent a watershed for the more extensive study of churches, sanctuaries, and other alternative sites hosting important works of art. This may certainly represent the most important contribution of this paper to the field of cultural heritage conservation. 

Further developments of this work will include the completion of the monitoring campaign. Diffusive passive samplers will be employed to study a wider range of gaseous pollutants (NO_x_, SO_2_, H_2_S, NH_3_, etc.) in order to gain a complete picture of the air quality within the Sanctuary. Moreover, continuous analyzers for the study of the same pollutants will be employed in order to evaluate the daily trends and variations. Finally, particulate matter gravimetric sampling will be performed in order to determine the chemical composition of the particles, which is extremely important in establishing the sources and the hazards linked to these pollutants.

## Figures and Tables

**Table 1:** The recommended microclimatic conditions in museum environments according to the D.M. 10 May 2001.

Parameter	Limit Values or Ranges
SO_2_	<0.4 ppb (vol)
NO_2_	<2.5 ppb (vol)
O_3_	1 ppb (vol)
PM10	20–30 µg m^−3^
Temperature	19–24 °C (painted wood) 6–25 °C (mural paintings)
Relative Humidity	45–65% (painted wood) 45–60% (mural paintings)
Illuminance	<150 lux (moderately light-sensitive exhibits and artifacts)

**Table 2:** The recommended microclimatic conditions in museum environments according to the UNI 10829:1999 technical standard.

Parameter	Limit Values or Ranges
Temperature	19–24 °C (painted wood) 10–24 °C (mural paintings)
Maximum daily temperature variation	1.5 °C (painted wood)
Relative Humidity	50–60% (painted wood) 45–55% (mural paintings)
Maximum daily relative humidity variation	4% (painted wood)

**Table 3:** Percentage of overrun days of the limits indicated in the UNI 10829:1999 technical standard.

Parameter	Datalogger	Overrun Days (Painted Wood)/%	Overrun Days (Wall Paintings)/%
Average daily temperature	DL 1	88	36
	DL 2	97	39
	DL 3	89	35
	DL 4	90	47
	DL 5	88	30
Average daily relative humidity	DL 1	51	76
	DL 2	50	79
	DL 3	29	53
	DL 4	61	67
	DL 5	36	61
Maximum daily relative humidity variation	DL 1	52	-
	DL 2	57	-
	DL 3	57	-
	DL 4	55	-
	DL 5	64	-
Maximum daily temperature variation	DL 1	3	-
	DL 2	1	-
	DL 3	9	-
	DL 4	4	-
	DL 5	15	-

**Table 4:** The illuminance values inside the Sanctuary.

Datalogger	Maximum Illuminance/lux	Minimum Illuminance/lux	Average Illuminance/lux
6	32.28	11.84	19.37
7	19.37	11.84	12.84

**Table 5:** The PM10, PM2.5, and PM1 average daily concentrations for the sampling site at the *Deposition*.

Date	PM10 Concentration/µg m^−3^	PM2.5 Concentration/µg m^−3^	PM1 Concentration/µg m^−3^
3 February 2021	19.4	15.4	14.0
3 April 2021	44.1	37.6	29.7
3 May 2021	62.6	35.9	28.6
3 September 2021	31.7	30.1	29.0
3 October 2021	22.7	20.6	19.5
14 March 2021	11.0	7.2	6.5
25 March 2021	29.2	24.7	22.9

**Table 6:** Nitrogen dioxide concentrations inside the Sanctuary compared with the outdoor values and recommended limits.

Sampling Site	Sampling Period	NO_2_ Indoor Concentration/µg m^−3^	NO_2_ Outdoor Concentration/µg m^−3^	NO_2_ Limit (D.M. 10 May 2001)/µg m^−3^
*Last Supper*	2 February 2021–23 March 2021	5.2	18.3	4.99
*Deposition*	23 March 2021–2 April 2021	6.7	17.7	4.89
*Last Supper*	14 December 2021–28 December 2021	15.0	45.3	5.08
*Deposition*	14 December 2021–28 December 2021	14.0	45.3	5.08
*Choir*	14 December 2021–28 December 2021	13.0	45.3	5.08

**Table 7:** The locations and monitored parameters of the dataloggers.

Datalogger	Location	Parameters Monitored
DL1	Ground floor, main lateral chapel, *Deposition*	Temperature, Relative Humidity
DL2	Ground floor, main lateral chapel, *Last Supper*	Temperature, Relative Humidity
DL3	First floor, ledge above main lateral chapel, *Last Supper*	Temperature, Relative Humidity
DL4	First floor, ledge above main lateral chapel, *Deposition*	Temperature, Relative Humidity
DL5	Second floor, dome	Temperature, Relative Humidity
DL6	Ground floor, main lateral chapel, *Last Supper*	Illuminance
DL7	Ground floor, main lateral chapel, *Deposition*	Illuminance

## Data Availability

Not applicable.

## Supplementary Materials

The following supporting information can be downloaded at: https://www.mdpi.com/article/10.3390/molecules28041615/s1, Figure S1: Average daily temperature trends in the Sanctuary; Figure S2: Average daily relative humidity trends in the Sanctuary; Figure S3: Particulate matter dimensional class distribution at the sampling site for *Deposition*. The ranges of the dimensional class are expressed in µm; Figure S4: Particulate matter dimensional class distribution at the sampling site for *Choir*. The ranges of the dimensional class are expressed in µm. Table S1: The PM10, PM2.5, and PM1 average daily concentrations at the sampling site for *Last Supper*; Table S2: The PM10, PM2.5, and PM1 average daily concentrations at the sampling site for *Choir*.
