# Supplementary material for: Preliminary Air Quality and Microclimatic Conditions Study in the Santuario della Beata Vergine dei Miracoli in Saronno (VA)
Source: Molecules. 2023 Feb 7;28(4):1615. doi: 10.3390/molecules28041615 (PMC9959446; doi:10.3390/molecules28041615)
Supplement: Supplementary file 1 [file molecules-28-01615-s001.zip › molecules-2151065-supplementary.pdf]

## Supplementary Materials

**Figure S1.** Average daily temperature trends in the Sanctuary.

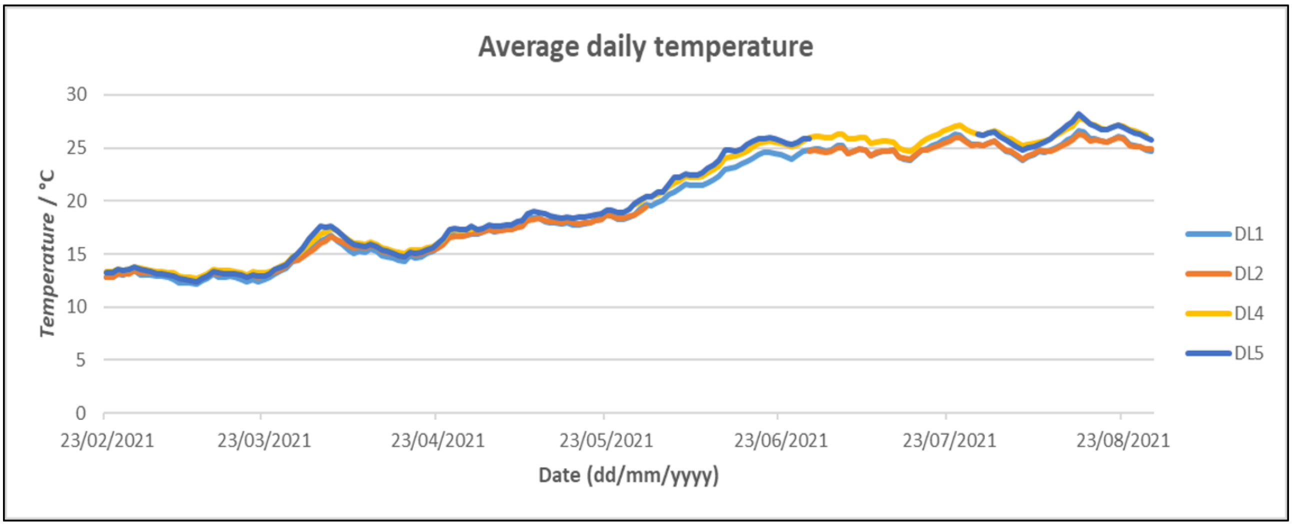


**Figure S2.** Average daily relative humidity trends in the Sanctuary.

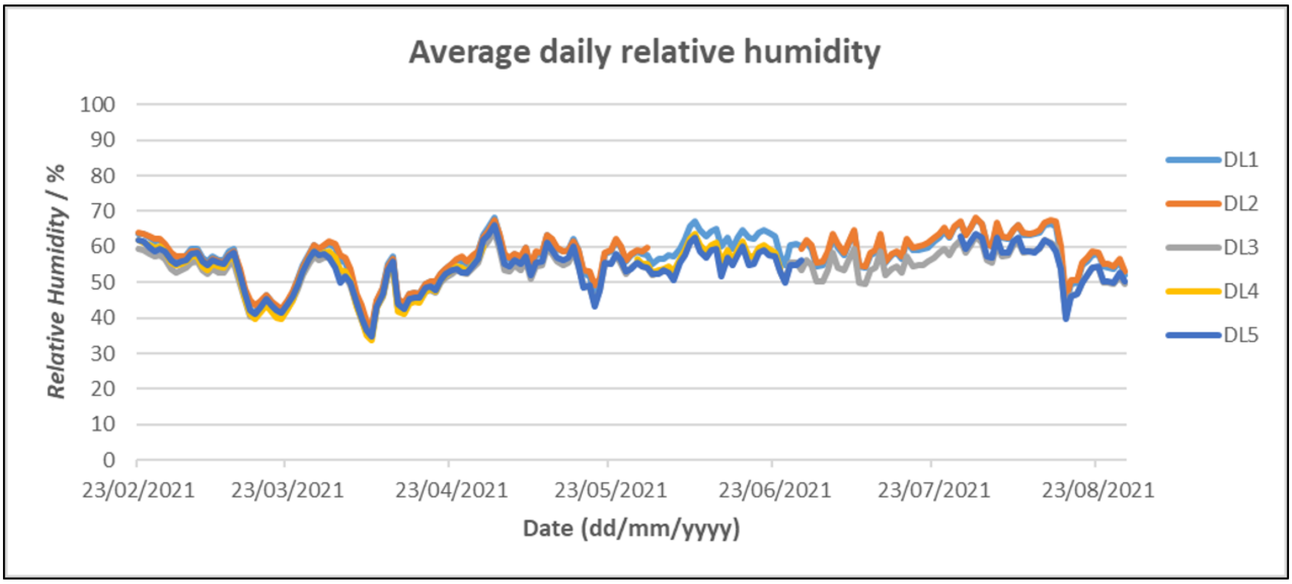

**Figure S3.** Particulate matter dimensional class distribution for the sampling site *Deposition*. The ranges of the dimensional class are expressed in µm.

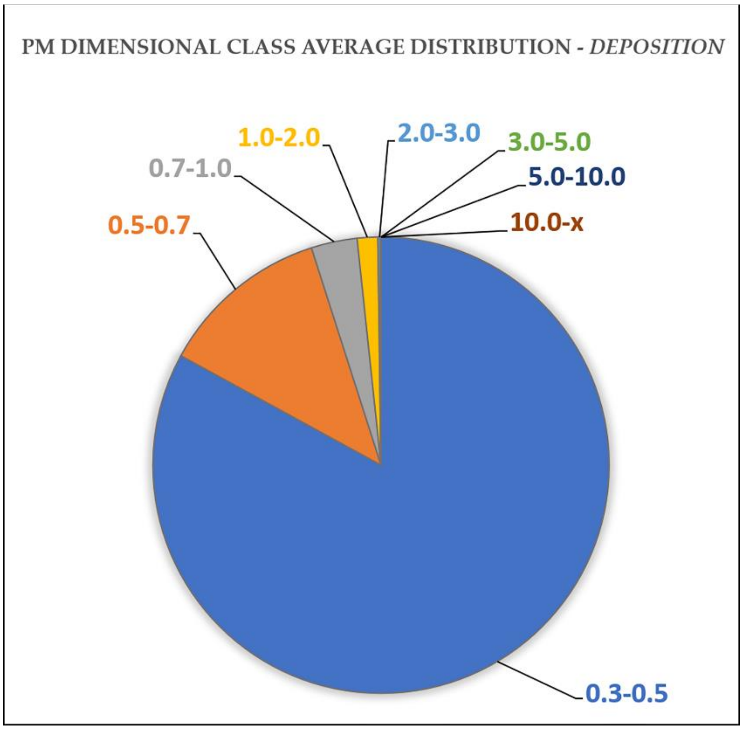


**Figure S4.** Particulate matter dimensional class distribution for the sampling site *Choir*. The ranges of the dimensional class are expressed in µm.

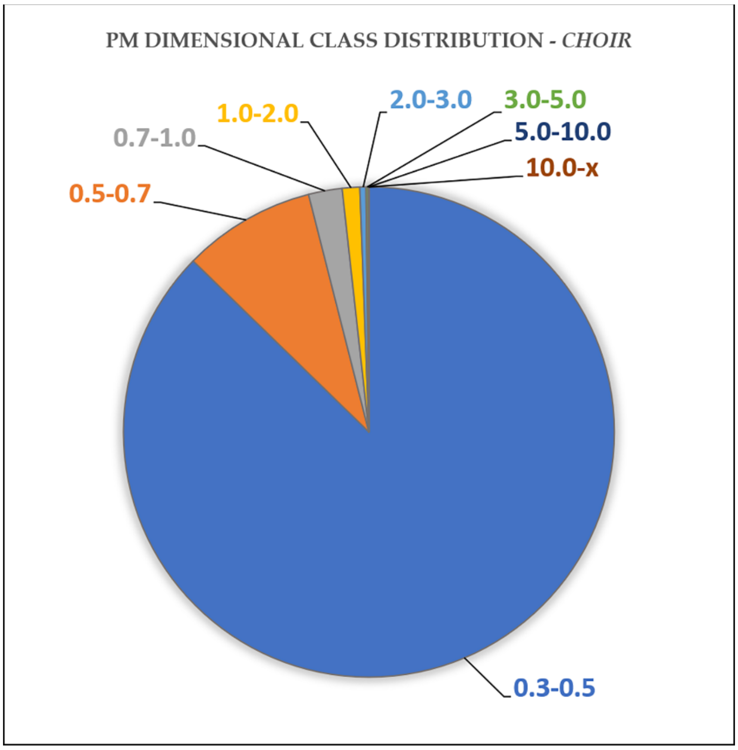

**Table S1.** PM10, PM2.5 and PM1 average daily concentrations for the sampling site *Last Supper*.

| Date | PM10 concentration / µg m$^{-3}$ | PM2.5 concentration / µg m$^{-3}$ | PM1 concentration / µg m$^{-3}$ |
|---|---|---|---|
| 03/25/2021 | 20.4 | 18.1 | 16.7 |
| 03/26/2021 | 19.4 | 15.9 | 13.5 |
| 03/27/2021 | 15.1 | 13.9 | 12.5 |
| 03/28/2021 | 40.8 | 38.3 | 32.0 |
| 03/29/2021 | 17.2 | 15.7 | 14.1 |
| 03/30/2021 | 21.6 | 19.7 | 18.1 |
| 03/31/2021 | 41.0 | 38.9 | 34.7 |
| 04/01/2021 | 38.5 | 36.0 | 31.5 |
| 04/02/2021 | 66.6 | 63.4 | 56.3 |
| 04/03/2021 | 31.5 | 28.9 | 23.5 |
| 04/04/2021 | 40.3 | 38.4 | 33.0 |
| 04/05/2021 | 15.6 | 14.2 | 11.6 |
| 04/06/2021 | 5.4 | 2.8 | 1.8 |
| 04/07/2021 | 2.8 | 2.0 | 1.6 |
| 04/08/2021 | 13.1 | 11.7 | 9.8 |
| 04/09/2021 | 9.7 | 6.3 | 4.2 |
| 04/10/2021 | 7.8 | 5.0 | 3.6 |
| 04/11/2021 | 7.9 | 6.9 | 6.3 |
| 04/12/2021 | 3.6 | 3.0 | 2.8 |
| 04/13/2021 | 3.1 | 2.2 | 1.7 |
| 04/14/2021 | 5.3 | 4.5 | 3.9 |
| 04/15/2021 | 4.7 | 3.7 | 3.1 |
| 04/16/2021 | 8.5 | 5.6 | 4.1 |
| 04/17/2021 | 10.7 | 9.7 | 8.9 |
| 04/18/2021 | 17.6 | 16.4 | 15.6 |
| 04/19/2021 | 16.6 | 13.6 | 12.4 |
| 04/20/2021 | 12.4 | 11.6 | 10.9 |
| 04/21/2021 | 7.2 | 6.2 | 5.7 |
| 04/22/2021 | 9.2 | 8.5 | 7.9 |
| 04/23/2021 | 17.3 | 16.3 | 15.2 |
| 04/24/2021 | 20.8 | 19.2 | 17.2 |
| 04/25/2021 | 45.6 | 42.9 | 36.7 |
| 04/26/2021 | 35.9 | 34.3 | 31.0 |
| 04/27/2021 | 11.3 | 10.4 | 9.3 |
| 04/28/2021 | 17.6 | 16.9 | 16.0 |
| 04/29/2021 | 21.5 | 21.0 | 20.2 |
| 04/30/2021 | 8.4 | 6.5 | 5.3 |
| 05/01/2021 | 12.2 | 11.1 | 10.2 |
| 05/02/2021 | 41.0 | 39.2 | 37.1 |
| 05/03/2021 | 11.2 | 10.2 | 9.4 |
| 05/04/2021 | 6.7 | 5.9 | 5.1 |
| 05/05/2021 | 4.9 | 4.2 | 3.3 |
| 05/06/2021 | 22.1 | 21.1 | 17.5 |
| 05/07/2021 | 50.0 | 49.1 | 44.7 |
| 11/23/2021 | 12.6 | 11.1 | 12.1 |
| 11/28/2021 | 10.0 | 7.7 | 7.0 |

**Table S2.** PM10, PM2.5 and PM1 average daily concentrations for the sampling site *Choir*.

| Date | PM10 concentration / µg $m^{-3}$ | PM2.5 concentration / µg $m^{-3}$ | PM1 concentration / µg $m^{-3}$ |
|---|---|---|---|
| 05/27/2021 | 5.2 | 4.0 | 3.0 |
| 05/28/2021 | 6.0 | 4.8 | 3.8 |
| 05/29/2021 | 10.8 | 9.5 | 7.5 |
| 05/30/2021 | 27.4 | 25.5 | 22.1 |
| 05/31/2021 | 16.1 | 15.0 | 13.6 |
| 06/01/2021 | 8.2 | 7.0 | 6.2 |
| 06/02/2021 | 8.3 | 7.3 | 6.7 |
| 06/03/2021 | 12.2 | 10.7 | 9.8 |
| 06/04/2021 | 8.3 | 6.9 | 6.1 |
| 06/05/2021 | 6.0 | 5.1 | 4.2 |
| 12/09/2021 | 27.1 | 25.9 | 23.7 |
| 12/12/2021 | 20.8 | 18.5 | 17.4 |
